# Lenses protecting against photosensitivity violate international driving regulations

**DOI:** 10.1002/epi.70170

**Published:** 2026-03-03

**Authors:** Lena Bender, Wolf Lagrèze, Martin Hirsch, Andreas Schulze‐Bonhage

**Affiliations:** ^1^ Epilepsy Center University Medical Center, University of Freiburg Freiburg Germany; ^2^ Neuro‐ophthalmology Section University Medical Center, University of Freiburg Freiburg Germany

To the Editors:

The insightful review on visually provoked seizures by Fisher et al.^1^ provides relevant and valuable information on a broad range of aspects related to photosensitivity and epilepsy. This includes potential real‐life trigger situations, such as a risk that may be posed by flickering light at frequencies associated with a high susceptibility to photoparoxysmal responses when driving along an avenue at dawn or sunset.

As a nonpharmacological treatment option to protect patients with photoparoxysmal responses, dark lenses and blue lenses—particularly the Zeiss Z1 blue lens—are discussed due to their ability to reduce or even abolish photosensitivity, as demonstrated in several studies (e.g., Takahashi and Tsukahara,^2^ Capovilla et al.^3^). In particular, the review notes that, when driving a car, tinted glasses with reduced light transmission are recommended. At the same time, however, the authors emphasize that lenses impairing the perception of orange traffic signals should be avoided.

We would like to highlight this latter point, as it is highly relevant when considering the use of tinted lenses in photosensitive patients who drive. Dark sunglasses are unsuitable for driving at sunset or during nighttime due to their reduced light transmission.^4^ In addition, the Z1 lens—and blue lenses in general—substantially alter the spectral composition of transmitted light by selectively attenuating longer wavelengths of the visible spectrum. This results in a marked reduction in the visibility of red and orange visual stimuli. Such stimuli are critical in traffic, particularly for the recognition of traffic lights as well as the brake lights and taillights of vehicles (Figure 1). Attenuation of red light intensity may delay stimulus recognition and consequently prolong reaction times while driving.

**FIGURE 1:**
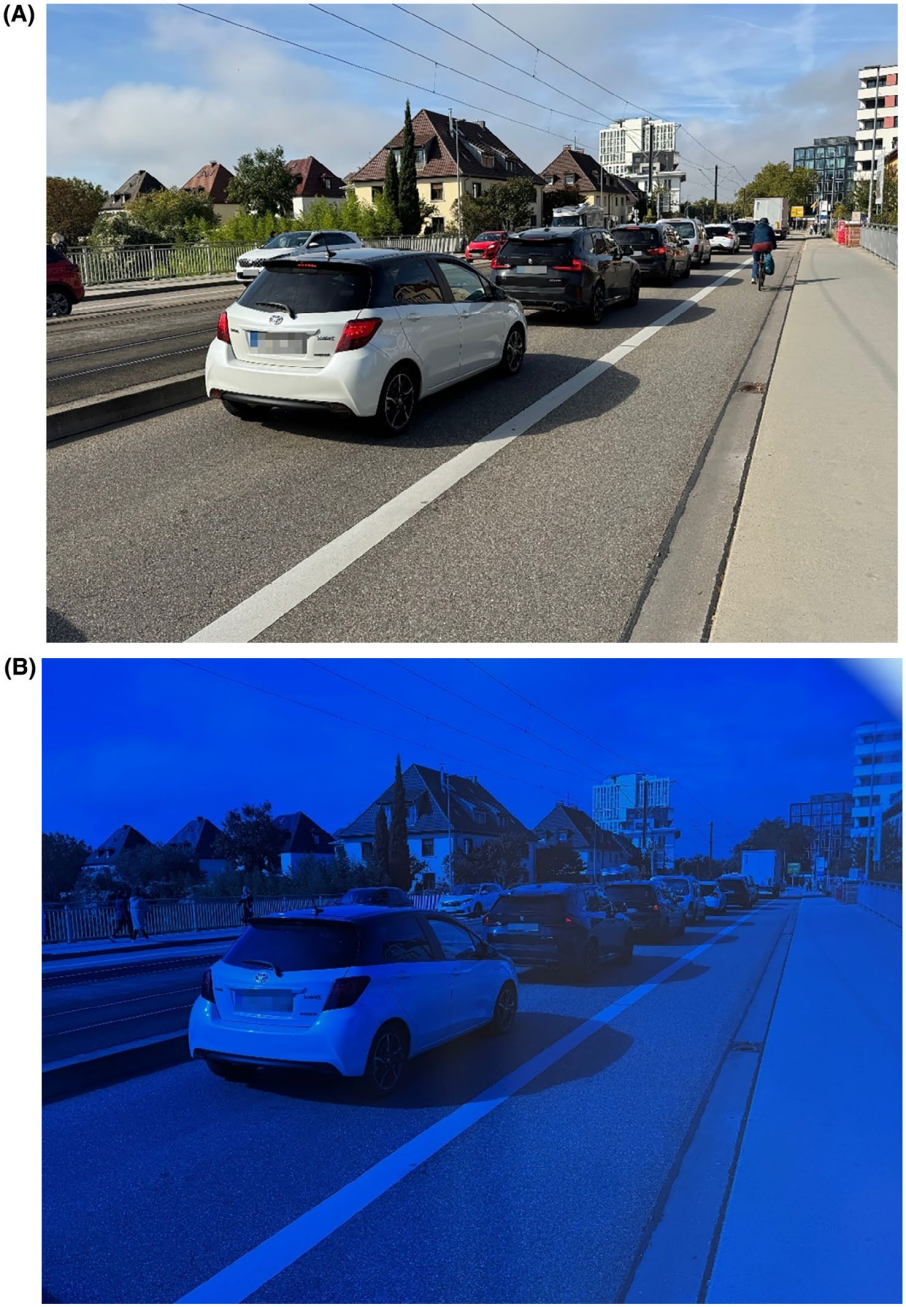
(A) Taillights of cars without tinted lens. (B) Taillights of cars with Z1 lens.

Therefore, although these lenses may exert beneficial effects in reducing photosensitivity, they may simultaneously increase the risk of traffic accidents. Accordingly, blue lenses—and especially Z1 lenses, which have the strongest evidence for suppressing photosensitivity—are not considered suitable for driving according to the International Organization for Standardization (ISO 12312‐1:2022).

Prescribing colored or dark lenses for patients with purely visually provoked seizures who may be legally permitted to drive once the triggering stimulus is suppressed therefore requires careful consideration of both their beneficial effects and potentially associated risks, as well as implications with regard to driving regulations.

## CONFLICT OF INTEREST STATEMENT

None of the authors has any conflict of interest related to this letter to disclose. We confirm that we have read the Journal's position on issues involved in ethical publication and affirm that this report is consistent with those guidelines.

## References

[1] Fisher RS , Wilkins A , Takahashi Y , Thio LL , Acharya J , Alkhaldi M , et al. Visually‐provoked seizures: consensus of the Epilepsy Foundation Working Group. Epilepsia. 2026;67(2):565–581. 10.1111/epi.18702 Epub 2025 Nov 24. 41283196

[2] Takahashi T , Tsukahara Y . Usefulness of blue sunglasses in photosensitive epilepsy. Epilepsia. 1992;33:517–521. 1592030 10.1111/j.1528-1157.1992.tb01702.x

[3] Capovilla G , Beccaria F , Romeo A , Veggiotti P , Canger R , Paladin F . Effectiveness of a particular blue lens on photoparoxysmal response in photosensitive epileptic patients. Ital J Neurol Sci. 1999;20:161–166. 10541598 10.1007/s100720050026

[4] Fisher RS , Acharya JN , Baumer FM , French JA , Parisi P , Solodar JH , et al. Visually sensitive seizures: an updated review by the Epilepsy Foundation. Epilepsia. 2022;63(4):739–768. 35132632 10.1111/epi.17175

